# Novel Molecular Signatures Selectively Predict Clinical Outcomes in Colon Cancer

**DOI:** 10.3390/cancers17060919

**Published:** 2025-03-07

**Authors:** Sarrah Lahorewala, Chandramukhi S. Panda, Karina Aguilar, Daley S. Morera, Huabin Zhu, Adriana L. Gramer, Tawhid Bhuiyan, Meera Nair, Amanda Barrett, Roni J. Bollag, Vinata B. Lokeshwar

**Affiliations:** 1 Departments of Biochemistry and Molecular Biology, Medical College of Georgia, Augusta University, Augusta, GA 30912, USA; sarrah.lahorewala@bcm.edu (S.L.); cpanda@augusta.edu (C.S.P.); kaaguilar@augusta.edu (K.A.); hzhu@arv-tech.com (H.Z.); 2 Department of Pathology Medical College of Georgia, Augusta University, Augusta, GA 30912, USA; dmorera@augusta.edu (D.S.M.); alangergramer@augusta.edu (A.L.G.); tbhuiyan@augusta.edu (T.B.); abarrett@augusta.edu (A.B.); rbollag@augusta.edu (R.J.B.); 3 Willaim J Brennan High School, San Antonio, TX 78253, USA; mairmeera708@gmail.com; 4 Bio-Repository Alliance of Georgia for Oncology (BRAG-Onc), Georgia Cancer Center, Medical College of Georgia, Augusta University, Augusta, GA 30912, USA

**Keywords:** colorectal cancer, prognostic markers, hyaluronic acid family, epithelial–mesenchymal transition, clinical outcome

## Abstract

**Background/Objectives**: The incidence of and deaths due to adenocarcinoma of the colon (COAD) and rectum (READ) are increasing, especially in younger adults. The early detection of metastatic COAD/READ by biomarkers could enable early intervention for improving outcomes. Hyaluronic acid (HA) family and associated epithelial–mesenchymal transition (EMT) markers promote COAD/READ progression. We evaluated the prognostic ability of HA-family and EMT markers in three COAD/READ cohorts. **Methods**: Transcript and/or protein expression of HA-family (HA, HAS-1, HAS-2, HAS-3, CD44, RHAMM, HYAL-1, and HYAL-4), and EMT markers (N-cadherin (N-Cadh), β-catenin, TWIST1, SNAI1, Slug, MMP-9, and Vimentin) were analyzed in normal and tumor specimens in two COAD cohorts from patients treated with surgery (cohort-1: *n* = 94; cohort-2: *n* = 68) and in the TCGA “COADREAD” cohort (*n* = 383). Expression levels were correlated with clinical outcomes (metastasis, overall survival, or recurrence-free survival) in univariate, multivariate, and Kaplan–Meier analyses. **Results**: In cohort-1, HA-family/EMT transcript levels were 2.5–11-fold higher in tumors compared to normal tissues. Hierarchical clustering based on combined transcript signatures CM-2 (HYAL-1 + N-Cadh) and CM-6 (HYAL-1 + HAS-2 + N-Cadh + SNAI1 + Slug + MMP-9) predicted and risk-stratified patients for metastasis (*p* ≤ 0.0001) with 80% and 89% accuracy, respectively. In validation cohort-2, elevated (3–10-fold) HA-family/EMT protein, HA and hyaluronidase levels were associated with metastasis (*p* < 0.0001). Combination protein marker signatures predicted and risk-stratified patients for metastasis (*p* ≤ 0.0001; >90% accuracy). Although in the TCGA COADREAD dataset CM-2 and CM-6 were independent predictors and risk-stratified patients for overall survival, the correlation with overall survival and disease-specific survival was significant only in patients with COAD (*p* ≤ 0.007) and not READ. **Conclusions**: The likely selectivity of HA-family/EMT signatures to predict clinical outcomes in patients with COAD could aid in their clinical translation and in designing HA-family/EMT-based targeted treatments for COAD.

## Simple Summary

The increasing incidence of adenocarcinoma of the colon (COAD) and the rectum (READ), especially in adults < 55 years and with COAD/READ being the second and fourth leading cause of cancer-related deaths in men and women, drives the need for the early detection of metastasis to enable early intervention. Hyaluronic acid (HA) family and associated epithelial–mesenchymal transition (EMT) markers promote COAD/READ metastasis. This study found that while the transcript and protein expression of fifteen HA-family/EMT markers were higher in tumor tissues and correlated with metastasis in three patient cohorts, combined marker signatures CM-2 and CM-6 independently predicted metastasis and overall survival with nearly 90% accuracy. Interestingly, the marker signatures were selective in predicting clinical outcomes only in patients with COAD and not READ, which warrants further validation. The selectivity of HA-family/EMT marker signatures for predicting prognosis in patients with COAD demonstrates their potential for clinical translation, and that treatments targeting HA-family/EMT could improve outcomes in patients with COAD.

## 1. Introduction

Among the 152,810 estimated new cases of adenocarcinoma of the colon (COAD) and the rectum (READ) in 2024, the rates of colorectal cancer (CRC) are increasing in young adults (age < 55 years) [1]. CRC was the fourth leading cause of cancer death in both men and women in the late 1990s, but is now the first in men and second in women [1]. The majority of CRC-related deaths are due to metastasis. More than 20% of patients with CRC have synchronous distant metastasis at diagnosis, and the five-year survival rate is about 15% [2,3,4,5]. The early detection and treatment of metastases could potentially improve patient survival, reinforcing the need to develop prognostic biomarkers that accurately predict metastasis and clinical outcomes, and subsequently guide therapy.

Hyaluronic acid (HA) is a large glycosaminoglycan consisting of repeating disaccharides D-glucuronic acid and N-acetyl-D-glucosamine. It is the only non-sulfated glycosaminoglycan. HA is an essential component of the extracellular matrix required for tissue hydration, osmotic balance, and the regulation of cellular functions, including cell adhesion, growth, motility, and invasion. The HA coat around the tumor cells protects them from immune surveillance. These functions are mediated through HA’s interaction with its receptors, CD44 and receptor for HA-mediated motility (RHAMM). HA levels are tightly regulated by the HA synthases (i.e., HAS-1, HAS-2, and HAS-3) and hyaluronidase (HAase) enzymes that degrade HA [6,7,8]. Several HA-family members have been implicated in tumor growth, metastasis, and angiogenesis [9,10,11,12]. HA-family members are also potential diagnostic and prognostic markers for a variety of cancers [13,14,15,16,17,18]. In CRC, members of the HA-family have been shown to promote tumor progression, invasion, and metastasis [19,20,21,22,23]. However, studies examining the biomarker potential of these molecules in CRC are lacking, and it is unknown if there is selectivity among the HA-family markers in predicting prognosis among patients with COAD versus READ.

Epithelial-to-Mesenchymal transition (EMT) is the acquisition of mesenchymal properties by epithelial tumor cells, allowing them to migrate to distant sites and form metastases. EMT is a hallmark of cancer metastasis, and the metastases promoting functions of EMT markers such as N-cadherin (N-Cadh), β-catenin, TWIST1, Snail (SNAI1), Slug (SNAI2), and MMP-9 are well established [24,25,26]. Upregulation of these genes is often a marker for poor clinical outcomes [27,28,29,30,31,32,33,34,35,36,37]. Some studies have reported the prognostic potential of these EMT markers in predicting clinical outcomes in patients with CRC [38,39,40,41,42,43,44,45,46,47,48]. There is compelling evidence to show that HA signaling drives EMT [49,50,51,52]. For example, we previously demonstrated that HA signaling induces EMT and that the expression of the HA-family correlates with EMT markers in bladder cancer [53]. However, the correlation between HA-family and EMT marker expression in CRC has not been examined.

In this study, we measured the expression of fifteen HA-family members (HA, CD44, RHAMM, HAS-1, HAS-2, HAS-3, HYAL-1, and HYAL-4), and EMT markers (N-Cadh, β-catenin, TWIST1, SNAI1, Slug, MMP-9, and Vimentin) in normal colon and tumor specimens from two COAD patient cohorts and correlated the levels with clinical outcomes. We also used The Cancer Genome Atlas (TCGA) COADREAD dataset, consisting of RNA-Seq and clinical data on 383 CRC specimens, to evaluate HA-family and EMT markers. Since READ accounts for about 30% of the total CRC cases, the TCGA dataset with 92 cases provides an opportunity to analyze the prognostic potential of any biomarker in patients with COAD versus READ. This dataset is particularly useful, as it includes a significant number of READ specimens showing selectivity in predicting clinical outcomes in patients with COAD versus READ. We also hypothesized that since HA signaling induces EMT, a combined HA-family/EMT signature(s) may be superior to individual molecules in predicting prognosis.

## 2. Materials and Methods

**Patients and Tissue Specimens:** Normal and primary tumor tissues were collected from patients undergoing partial colectomy at the Medical College of Georgia, Augusta University (MCG-AU). All specimens were obtained based on their availability for research through the Bio-Repository Alliance of Georgia for Oncology (BRAG-ONC) under a protocol approved by the AU institutional review board (protocol # 611107). Cohort-1 consisted of 27 normal colon and 67 tumor specimens and cohort-2 included 11 normal and 57 tumor specimens. Tumor specimens were obtained at surgery. In both cohorts, tumor specimens included only 2 READ samples. In this cohort, patients positive for metastasis had disseminated disease. The TCGA COADREAD dataset was accessed through the UCSC Xena Browser [54]. The TCGA dataset contains 383 patients on whom transcript expression data are available, along with demographic and pathologic parameters and clinical outcomes in terms of survival. The dataset does not contain information regarding time to metastasis, and metastasis data were missing for 77 patients. Patient characteristics for the clinical cohorts and the TCGA datasets are described in Supplementary Table S1.

**Reverse transcription quantitative PCR (RT-qPCR):** RNA was extracted from approximately 30 mg of tissue using the RNeasy kit (Qiagen, Germantown, MD, USA). RNA was then reverse transcribed (RT; iScript; Bio-Rad Laboratories, Hercules, CA, USA) and subjected to RT-qPCR using gene-specific primers; sequences are provided in Supplementary Table S2. The normalized transcript levels for each gene were calculated as (100/2^ΔCq^); ΔCq = Cq(transcript) − Cq(β actin) [55,56]. Each specimen was analyzed at least in duplicate in each assay. The average normalized value from each specimen was used when calculating the data for each marker in the specimen cohort.

**Hierarchical Cluster Analysis**: The expression data of each marker, calculated from the Z-score, were used for generating the heatmap [57]. The Z-score for each specimen was calculated from the mean ± SD expression levels of a marker in all specimens in a cohort.

**Immunohistochemistry (IHC):** Normal and tumor specimens were stained for HA-family and EMT markers using an IHC procedure described previously [13,58]. Antibodies and dilutions are provided in Supplementary Table S3. Stained slides were independently graded by two authors (S.L. and V.B.L.) in a blind fashion, for intensity and the areas, as described before [15,59]. Intensity scores in all areas were added and then divided by 100 to obtain a staining score for the entire specimen. The staining scores of the two authors were then averaged to obtain the final score.

**Immunoblot analyses:** Freshly frozen normal colon and tumor tissues (30–50 mg) were homogenized on ice in ice-cold RIPA buffer (pH 7.4), and then clarified via centrifugation at 14,000 rpm for 20 min. Tissue homogenates were subjected to immunoblotting for HA-family and EMT markers; all antibodies used are described in Supplementary Table S3. The samples were normalized based on total protein and actin; protein concentration was measured using the DC™ Protein Assay from Bio-Rad (Hercules CA). In each blot, all sample lanes were run on the same gel with the same exposure time.

**HA and HAase Tests**: Freshly frozen normal colon and tumor tissues were homogenized and centrifuged as described above in 5 mM Hepes buffer (pH 7.2) with protease inhibitors. The enzyme-linked immunosorbent assay-like assays that measure the HA and HAase activity levels in tissues were performed and the levels were normalized to total protein levels, as described before [60,61,62].

**Statistical Analysis:** JMP Pro 17 and GraphPad Prism software version 10 were used for the analyses. The differences in the biomarker levels between normal and tumor tissues were evaluated using the Mann–Whitney U test, as the data were non-normally distributed according to the Shapiro–Wilk test. Similarly, within each cohort, the differences in the levels of biomarkers with respect to T-stage, lymph node involvement (i.e., N-stage), metastasis, lymphovascular invasion (LVI), perineural invasion (PNI), and overall survival (OS) were also compared using the Mann–Whitney U test. All reported *p*-values in this study are two-tailed. Combined marker signatures were calculated by performing logistic regression analysis and including the indicated markers. The expression of the signature for each specimen/patient was calculated using the intercept and the coefficient for each marker that was included in the model, as described before (Supplementary Table S4) [55,56]. The logistic regression single-parameter model (univariate analysis) was used to determine the association of clinical parameters and the marker expression with metastasis and OS. Based on Youden’s index from the ROC curve, the optimal cut-off values were calculated to compute sensitivity and specificity. The Cox Proportional Hazards Model (multivariate analysis) was used to determine which clinical demographic and pathologic parameters and/or biomarker signatures were significant in predicting clinical outcomes. Kaplan–Meier plots with log-rank statistics were prepared for combined marker signatures in each cohort.

## 3. Results

### 3.1. Differential Expression of HA-Family and EMT Markers in Normal and CRC Tissues

**Figure 1:**
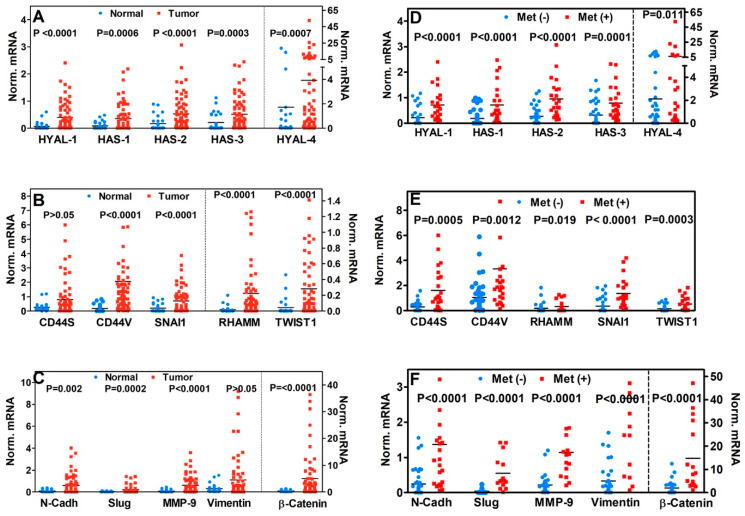
Differential transcript expression of 15 HA-family and EMT markers in Clinical Cohort-1. Scatter plot for each marker, where each dot represents a single specimen. Line represents the median marker level for each group. *p*-values were two-tailed and calculated using the Mann–Whitney test. (**A**–**C**) Comparison of marker levels between normal (*n* = 27) and CRC (*n* = 67) specimens. (**D**–**F**) Comparison of marker levels between non-metastatic (*n* = 43) and metastatic (*n* = 24) specimens.

We measured the expression of HA-family and EMT marker transcripts in cohort-1, which consisted of normal colon tissues (*n* = 27) and tumor specimens from 65 patients with COAD and two patients with READ. The eight HA-family members included in the analyses were HAS-1, HAS-2, HAS-3, HYAL-1, HYAL-4, CD44 Standard isoform (CD44S), CD44 Variant isoforms (CD44V), and RHAMM. The EMT markers analyzed were N-Cadh, β-catenin, TWIST1, SNAI1, Slug, MMP-9, and Vimentin. The median transcript levels of HA-family were elevated 2–30-fold in tumor tissues compared to normal colon tissues (Figure 1A,B). The same data are presented as box plots for all markers (Supplementary Figure S1). Similarly, the expression of EMT markers was also elevated 2–38-fold in tumor tissues compared to normal colon tissues (Figure 1B,C). The differences in the marker levels between tumor and normal specimens were statistically significant for all markers except for CD44S and Vimentin (Figure 1A–C).

### 3.2. Association of HA-Family and EMT Markers with Pathological Parameters and Clinical Outcomes

We compared the expression of HA-family and EMT markers with tumor stage, lymph node involvement, LVI, and PNI. Slug and Vimentin levels were significantly increased in patients with T-stage ≥ T3, while patients who had lymph node metastasis had significantly higher HAS-2, SNAI1, TWIST1, N-Cadh, Slug, and MMP-9 (Supplementary Table S5). None of the markers were significantly associated with LVI or PNI (Supplementary Table S5).

Of the 67 patients in the cohort, 24 developed metastasis during follow-up (Supplementary Table S1). Consistent with the functions of the HA-family and EMT markers in promoting metastasis, the levels of all markers increased by 2.5–14-fold in tumor specimens from patients who developed metastasis compared to those who did not, and this increase was statistically significant in each comparison (Figure 1D–F). Although the cause of mortality was unknown, ten patients died during follow-up, i.e., 85% OS. The transcript levels of N-Cadh were significantly associated with OS (Supplementary Table S5). In univariate analysis, except PNI, none of the demographic or pathological parameters were associated with metastasis (Table 1). All markers except HYAL-4, CD44V, and RHAMM were significantly associated with metastasis (Table 1). Furthermore, HYAL-1, HAS-2, SNAI1, N-Cadh, Slug, and MMP-9 predicted metastasis with > 80% efficacy; sensitivity: 79% to 91%; and specificity: 70% to 95% (Table 1).

### 3.3. Association of Novel HA-Family/EMT Signatures with Prognosis

**Figure 2:**
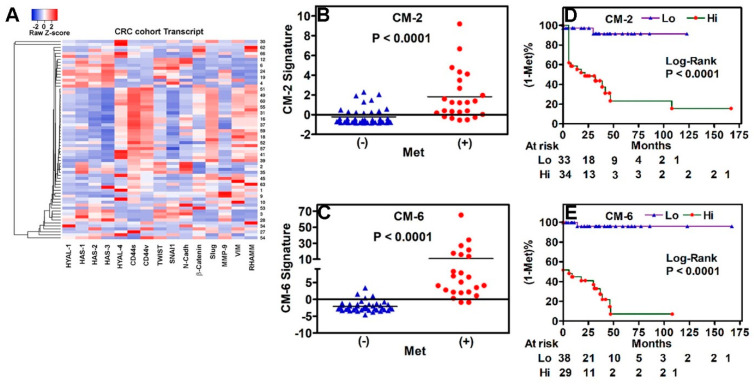
Association of the HA-family/EMT signatures with clinical outcome in cohort-1. (**A**) Hierarchical clustering of tumor specimens in cohort-1 by marker expression. The expression data of each marker, calculated using the Z-score, were used for generating the heatmap. (**B**,**C**) HYAL1 + N-Cadh (CM-2) and HYAL-1 + HAS-2 + N-Cadh + Slug + SNAI1 + MMP-9 (CM-6) signature expression in tumor specimens was stratified by metastasis. The line represents the Mean value of the signature value in each group. *p*-values were two-tailed and calculated using the Mann–Whitney test. (**B**) CM-2. (**C**) CM-6. (**D**,**E**) Kaplan–Meier plots for metastasis; risk was stratified by CM-2 (**D**) and CM-6 (**E**) signatures. The low and high levels of each signature were stratified by the Youden index, calculated from the ROC curve. *p*-value Log-Rank test.

Although six out of the fifteen HA-family and EMT markers predicted metastasis with >80% efficacy, higher sensitivity resulted in lower specificity (~70%). Therefore, we evaluated whether a combination of markers was superior in predicting prognosis than individual markers. We next performed unsupervised hierarchical cluster analysis to identify which EMT and HA markers have similar expression patterns across all specimens in cohort-1, and if such a pattern could be used to develop predictive biomarker signatures. Hierarchical clustering revealed a similar pattern of expression for HYAL-1, HAS-1, HAS-2, N-Cadh, MMP-9, SNAI1, and Slug transcripts (Figure 2A). Therefore, we assessed whether marker combinations within this sub-group would generate signatures that were independent predictors of metastasis with high accuracy.

We found that the levels of combination signatures CM-2 (HYAL-1 + N-Cadh) and CM-6 (HYAL-1 + N-Cadh + HAS-2 + SNAI1 + Slug + MMP-9) were elevated 5–8-fold in tumor specimens from patients who had or developed metastasis during follow-up compared to those who did not (*p* < 0.0001; Figure 2B,C). In both univariate and multivariate analyses, the signatures were significant predictors of metastasis (Table 1). Kaplan–Meier plots showed that higher CM-2 or CM-6 levels stratified patients for higher risk of metastasis (Log-Rank: *p* < 0.0001; Figure 2D,E).

### 3.4. Protein Expression Validates Transcript Signatures as Prognostic Markers

**Figure 3:**
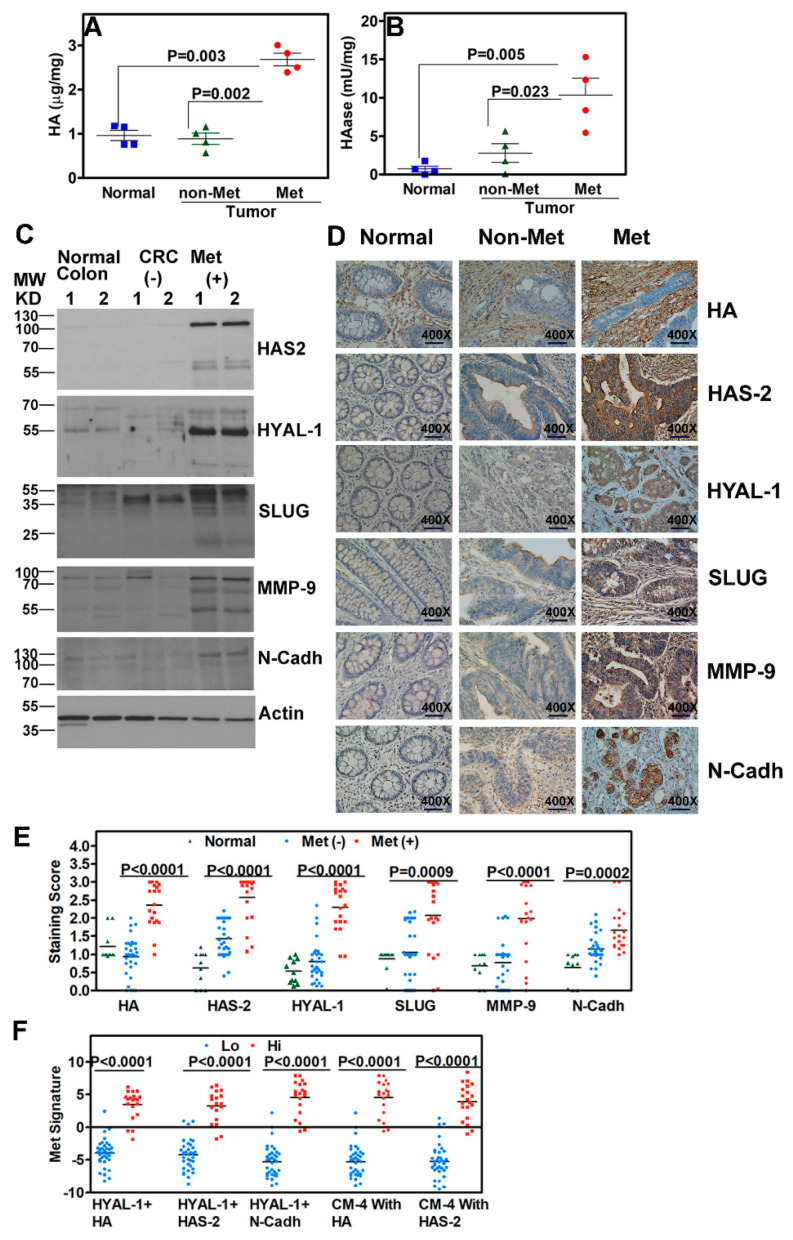
HA and HAase levels and the expression of HA-family/EMT protein markers and the signatures in clinical cohorts 1 and 2. (**A**,**B**) HA (**A**) and HAase (**B**) levels were measured in normal colon and tumor tissue extracts using HA and HAase tests. The levels were normalized to total protein. Each dot represents HA or HAase levels in a tissue. (**C**) Immunoblot analysis of normal colon and tumor specimens (*n* = 2/category) for the indicated proteins; actin: loading control. Tumor specimens were from patients who either developed or did not develop metastasis (Met) during follow-up. The uncropped blots and row data are shown in Figure S2 (**D**) Normal colon and tumor specimens from the metastasis and non-metastasis groups were stained via immunohistochemistry for individual markers. Representative images are shown; magnification: 400×. (**E**) Quantification of the IHC staining scores in clinical cohort-2 for tumor specimens. Tumor specimens were stratified based on the development of metastasis. (**F**) Signature expression by combining either two (CM-2) or four (CM-4) markers in tumor specimens stratified based on the development of metastasis. Note the markers were combined based on a combination reaching statistical significance.

We used several approaches to validate the upregulation of HA-family and EMT markers in COAD. We measured HA and hyaluronidase activity in normal colon and tumor tissues using the HA and HAase tests [60,61,62]. HA levels in tumor tissues from patients who developed metastasis were 3-fold higher compared to normal colon tissues and in tumor tissues from patients who did not develop metastasis (*p* = 0.003; Figure 3A). Similarly, HAase levels were 10-fold and 3.7-fold higher in tumor tissues from patients who developed metastasis compared to normal colon tissues and in tumors from patients who did not develop metastasis, respectively (*p* = 0.005; Figure 3B).

We next performed immunoblotting to qualitatively determine whether the markers in the CM-6 signatures were expressed at the protein level and at the predicted molecular size in normal and tumor specimens. Figure 3C shows the higher expression of the markers in tumor specimens from patients who developed metastasis during follow-up compared to patients who did not develop metastasis and normal colon specimens.

**Figure 4:**
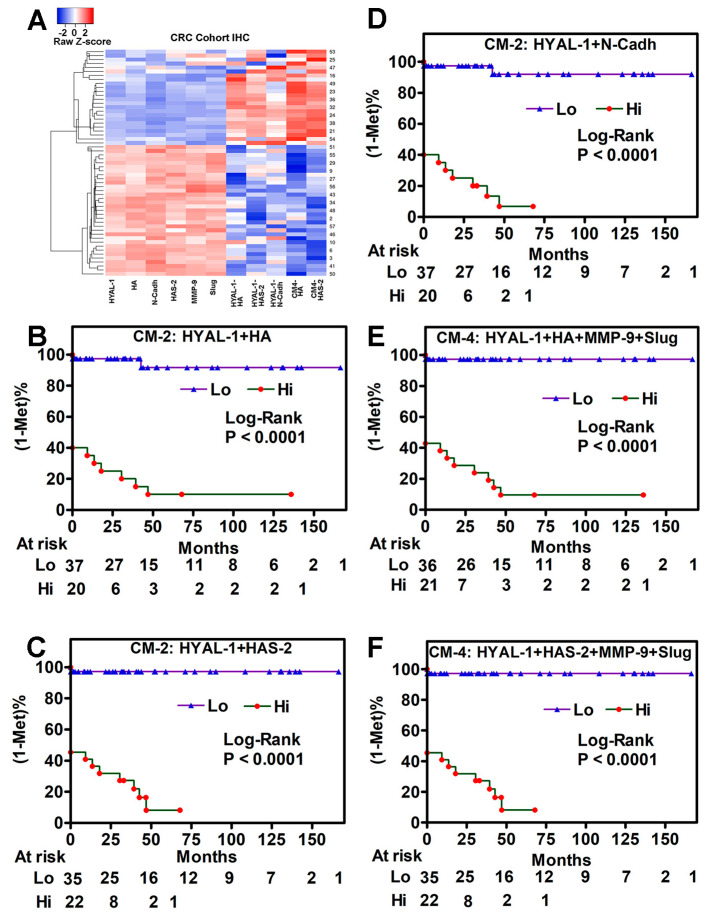
Association of HA-family/EMT protein biomarker signatures with clinical outcomes. (**A**) Hierarchical clustering of tumor specimens in clinical cohort-2 by IHC staining scores for each marker and the indicated CM-2 and CM-4 expression signatures. Expression data of each marker, calculated from the Z-score, were used for generating the heatmap. (**B**–**F**) Kaplan–Meier plots of metastasis risk stratified by the indicated HA-family/EMT signatures. The low and high levels of each signature were stratified by the Youden index calculated from the ROC curve. *p*-value Log-Rank test.

At the protein level, SNAI1 (Snail) antibodies did not yield a specific signal, and therefore, SNAI1 was not pursued as a marker at the protein level (Figure 3C). We next used IHC to validate HYAL-1, HAS-2, N-Cadh, Slug, MMP-9, and HA expression and their combinations in cohort-2. IHC showed that the five markers, HYAL-1, HAS-2, N-Cadh, Slug, and MMP-9 included in the CM-6 signature, were upregulated in tumor tissues from patients who developed metastasis compared to normal colon tissues and in tumors from patients who did not develop metastasis (Figure 3D). As an extracellular matrix component, while HA was localized in the stromal compartment, HAS-2, HYAL-1, Slug, MMP-9, and N-Cadh were expressed in tumor cells (Figure 3D). While normal colon tissues showed very low expression of these markers, their expression was significantly elevated in tumor specimens from patients who had or developed metastasis during follow-up compared to those negative for metastasis (Figure 3E,F). Hierarchical clustering showed the grouping of all five markers, confirming the close functional association of HA-family and EMT markers, both of which are elevated in COAD (Figure 4A).

In univariate analysis HA, HAS-2, HYAL-1, Slug, MMP-9, and N-Cadh staining in tumor tissues significantly correlated with metastasis (*p* ≤ 0.002 in each case; Table 2). In efficacy analysis, all markers showed high sensitivity and specificity, with HA and HYAL-1 demonstrating both sensitivity and specificity ≥ 90% (Table 2).

In all signatures with two marker combinations, the combination of HYAL-1 with HA, HAS-2, or N-Cadh significantly improved the prediction of metastasis, with sensitivity reaching 100% and 92–95% specificity (Table 2). Since HAS-2 synthesizes HA, when testing the predictability of a signature that includes all markers, we included either HA or HAS-2 in the combination. The inclusion of N-Cadh staining levels in the model did not improve the prediction of metastasis by the biomarker signatures. Therefore, we evaluated the prognostic ability of two, four-marker signatures (CM-4), HYAL-1 + HA + Slug + MMP-9 and HYAL-1 + HAS-2 + Slug + MMP-9. In univariate and multivariate analyses, both CM-4 signatures significantly predicted metastasis and were independent prognostic signatures. Both CM-4 signatures had the highest sensitivity (100%) and specificity (92–95%). Interestingly, the CM-4 signatures had the same accuracy in predicting metastasis as the HYAL-1+HAS-2 signature, suggesting that HYAL-1 and HAS-2 staining inferences are strongly associated with metastasis (Table 2). Kaplan–Meier plots showed that all three CM-2 and both CM-4 signatures risk-stratified patients for metastasis; high signature levels indicated higher risk for metastasis (*p* < 0.0001 for each signature; Figure 4B–F).

### 3.5. Association of Marker Levels with Prognosis in the TCGA COADREAD Dataset

**Figure 5:**
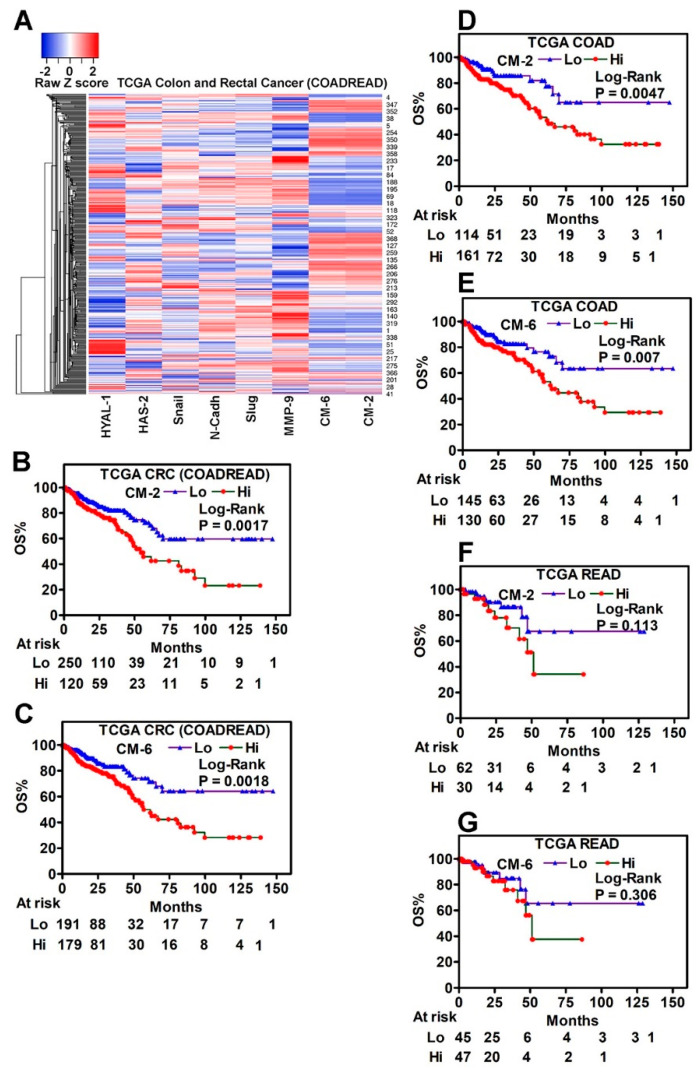
Differential expression of HA-family, EMT markers, and HA-family/EMT signatures in the TCGA COADREAD dataset. (**A**) Hierarchical clustering of the specimens in the TCGA COADREAD dataset (n-383) by the expression of the six markers (HYAL-1, HAS-2, N-Cadh, SNAI1, Slug, and MMP-9) expression. Expression data of each marker, calculated from the Z-score, were used for generating the heatmap. (**B**,**C**) Kaplan–Meier plots for overall survival (OS); risk stratified by CM-2 (HYAL1 + N-Cadh) (**B**) and CM-6 (HYAL-1 + HAS-2 + N-Cadh + Slug + SNAI1 + MMP-9 (**C**) signatures. (**D**,**E**) The expression of CM-2 and CM-6 signatures was analyzed in the COAD subset: Colon adenocarcinoma, *n* = 245; colon mucinous adenocarcinoma, *n* = 37. Kaplan–Meier plots for overall survival (OS); risk stratified by CM-2 (**D**) and CM-6 (**E**) signatures. (**F**,**G**) The expression of CM-2 and CM-6 signatures was analyzed in the READ subset: Rectal adenocarcinoma, *n* = 87; rectal mucinous adenocarcinoma, *n* = 5. (**B**–**G**) The low and high levels of each signature were stratified by the Youden index calculated from the ROC curve. *p*-value Log-Rank test.

In both clinical cohorts, all specimens except two READ specimens were COAD. To determine if the prognostic ability of the marker signatures was different in COAD and READ, we analyzed the COADREAD dataset, which consists of histologically confirmed 282 COAD and 92 READ specimens, with transcript data available for all HA-family and EMT markers. We used the entire dataset and COAD and READ subgroups to validate the CM-2 (HYAL-1 + N-Cadh) and CM-6 (HYAL-1 + N-Cadh + HAS-2 + SNAI1 + Slug + MMP-9) transcript signatures, as these signatures showed independent prognostic capabilities in predicting clinical outcomes in cohort-1 (Figure 1 and Figure 2; Table 1). Hierarchical clustering showed some grouping for the six markers in the COADREAD dataset; however, the grouping of specimens with respect to CM-2 and CM-6 was more prominent (Figure 5A). In the COADREAD dataset, the time to metastasis parameter is missing; however, the OS and DSS data are available.

In univariate and multivariate analyses, age, pathological parameters (stage, lymph node, and M-stage), and both combination signatures significantly predicted OS (Table 3).

Kaplan–Meier plots showed both CM-2 and CM-6 signatures risk-stratified patients in the COADREAD dataset for OS. As was observed in clinical cohort-1, tumors with high CM-2 or CM-6 levels were associated with lower OS for patients (Figure 5B,C). In the COAD subgroup, both in univariate and multivariate analyses, CM-2 and CM-6 significantly predicted OS (Supplementary Table S6). As observed in the COADREAD dataset, in the COAD cohort, higher CM-2 and CM-6 levels in primary tumors significantly predicted lower OS for patients during follow-up (Figure 5D,E). However, in the READ subgroup, neither in univariate nor in multivariate analyses, CM-2 and CM-6 signatures predicted clinical outcomes (Supplementary Table S7). Kaplan–Meier plots echoed these findings, as the CM-2 and CM-6 signatures failed to risk-stratify patients with READ for the prediction of OS (Figure 5F,G).

The selectivity of CM-2 and CM-6 signatures was more distinct in the prediction of disease-specific survival (DSS). While CM-2 and CM-6 were not significant predictors of DSS in the entire TCGA COADREAD dataset, these signatures risk-stratified patients for the prediction of DSS (Supplementary Figure S3A–D). CM-6 was also an independent predictor of DSS in patients with COAD (*p* = 0.0418). Both signatures failed to predict DSS in patients with READ (Supplementary Figure S3E,F).

## 4. Discussion

Locally advanced CRC is generally curable, with a 5-year survival rate as high as 90%. However, the prognosis for patients with metastatic CRC is poor [1,2,4,5]. As metastatic CRC remains uncurable, prognostic biomarkers that could aid in the early detection of metastasis and guide therapy are a critical requirement to decrease metastatic CRC-related mortality. The key findings of our study are two-fold. First, unique combinations of HA-family and EMT markers are potentially independent and accurate predictors of clinical outcomes in patients with COAD. The second rather unexpected finding is that the prognostic capability of these combined markers is selective for COAD and not for READ.

Some members of the HA-family and EMT markers have been individually evaluated in CRC [22,26,39,40,46,47,48]. However, our study is the first to examine the prognostic utility of fifteen members of this group of molecules in three cohorts both at the transcript and the protein levels. Furthermore, the combination of marker signatures discovered and validated in our study accurately predicts clinical outcomes in terms of metastasis and survival in patients with COAD. Contrary to the earlier findings on the prognostic ability of certain HA-family and EMT markers (e.g., CD44, β-catenin, VIMENT1), we found their prognostic ability to be limited or modest in comparison to the other members of these families, and that their inclusion in the combinations diminished the prognostic efficacy of the signatures [38,39,42].

In this study, the test and validation cohorts reveal similarities and differences among the markers that can be combined as prognostic signatures when evaluated as transcript or protein signatures. For example, while only CM-2 (HYAL-1 + N-Cadh) and CM-6 (HYAL-1 + HAS-2 + N-Cadh + Slug + SNI1 + MMP-9) transcript signatures were independent prognostic indicators with high efficacy in predicting metastasis and survival, several two-marker combinations of the HA-family/EMT markers, when evaluated by IHC, accurately predicted prognosis. In comparison with the CM-6 transcript signature, protein expression of N-Cadh did not improve the accuracy of the combination signature, and SNAI1 IHC was not specific. Therefore, the CM-4 protein signature (HYAL-1 + HAS-2 + Slug + MMP-9) and the CM-6 transcript signature (HYAL-1 + HAS-2 + N-Cadh + Slug + SNI1 + MMP-9) were independent prognostic indicators and had comparable efficacy. Nevertheless, our study shows that when using the marker signatures identified in one study (or one cohort) for predicting prognosis in another study, it is critical to use the same material (transcript or protein) and technique. The potential for the clinical translation of either the CM-4 protein signature or CM-6 transcript signature will depend on multiple factors. For example, the limitations of any IHC-based protocol include antibody availability, particularly if the antibody is polyclonal, tissue quality, and variation in IHC methodology. A PCR-based method would be more consistent, if the tissue quality is maintained. Nevertheless, we have recently reported the consistency of a qPCR-based assay for evaluating prognostic markers using formalin-fixed tissues, which could address the tissue quality and RNA stability issues [63].

Although COAD and READ are lumped together as CRC, 70% or more CRC cases are COAD. Furthermore, there are obvious differences in the molecular carcinogenesis, histopathology, surgical topography, and surgical and clinical management of COAD and READ [64]. Few studies have investigated the prognostic significance of HA-family/EMT markers or other markers in COAD versus READ. The TCGA dataset, with a relatively large collection of both COAD (*n* = 282) and READ (*n* = 92), allows for the analysis of markers separately in these two cohorts. At the molecular level, our study reveals differences in COAD and READ with respect to the HA-family and EMT markers. Both CM-2 and CM-6 transcript signatures were prognostic signatures for predicting OS and DSS in patients with COAD, but had no prognostic significance for READ. One limitation of the study is that the number of positive events (i.e., death) are low in the TCGA READ subgroup. Nevertheless, our study’s findings have broader implications, because the prognostic selectivity of HA-family and EMT markers may be reflective of the molecular mechanisms that drive disease progression in these two cancer types that arise in anatomically proximal tissues.

## 5. Conclusions

The major clinical implication of this study is that the HA-family/EMT signature not only predicted clinical outcomes, but did so with high accuracy. Utilization of this signature could allow for the identification of patients at high-risk of developing metastasis, and those who may benefit from aggressive treatment and surveillance. We have demonstrated the biomarker potential of this signature at both the transcript and protein levels using high-throughput assays. Taken together, this suggests that the HA-family/EMT signature could potentially be developed into a clinical assay for the early detection of metastasis. Whether and how the HA-family induces EMT in CRC remains to be elucidated. Further investigation into the functional significance of these markers is warranted to better understand their role in CRC pathogenesis and to develop HA-family/EMT-based targeted treatments.

## Figures and Tables

**Table 1:** Univariate and multivariate analysis of clinical parameters and transcript levels to predict metastasis in cohort-1. Logistic regression analyses were performed on demographic, clinical parameters, and each biomarker. Biomarkers showing area under the curve > 0.8 on the ROC curves were combined. Combinations HYAL-1 + N-Cadh + HAS2 + SNAI1 + Slug + MMP-9 (CM-6) and HYAL-1 + N-Cdh (CM-2) reached significance in univariate analysis. The Cox Proportional Hazards Model evaluated the ability of clinical parameters and combined markers to associate with metastasis. The model included age, sex, T-stage, N-stage, perineural invasion (PNI), lymphovascular invasion (LVI), and CM-2 or CM-6. Parameters reaching significance are shown. NA: Not applicable.

Univariate Analysis					
Parameter	*p*-Value	Unit OR; 95% CI	AUC	Sensitivity	Specificity
Age	0.0576	NA	NA	NA	NA
Sex	0.7023	NA	NA	NA	NA
T-stage (< vs. ≥3)	0.0573	NA	NA	NA	NA
N-Stage (+ vs. −)	0.0548	NA	NA	NA	NA
M-stage (+ vs. −)	NA	NA	NA	NA	NA
PNI (+ vs. −)	0.0261	3.7; 1.2–11.6	NA	NA	NA
LVI (+ vs. −)	0.0944	NA	NA	NA	NA
HYAL-1	0.0017	8.3; 2.2–31	0.818	83.33	72.1
HAS-1	0.0034	6.67; 1.9–23.7	0.793	75	74.4
HAS-2	0.0005	10.1; 2.7–37.3	0.849	87.5	69.8
HAS-3	0.0136	3.2; 1.3–7.9	0.771	91.7	65.1
HYAL-4	0.119	NA	NA	NA	NA
CD44S	0.0031	3.61; 1.5–8.5	0.762	71	90.7
CD44V	0.155	NA	NA	NA	NA
TWIST1	0.0036	11.1; 2.2–55.7	NA	NA	NA
SNAI1	0.0011	4.0; 1.7–9.1	0.835	87.5	74.4
N-Cadh	0.0011	5.3; 2.0–14.4	0.843	87.5	70
β-Catenin	0.0074	1.2; 1.0–1.4	0.787	75	76.4
Slug	0.0003	557,536; 1023–2.807 × 10^9^	0.891	79.2	90.7
MMP-9	0.0002	13.1; 3.4–50.2	0.909	91.7	81.4
Vimentin	0.0072	2.1; 1.2–3.7	0.773	54.2	95.3
RHAMM	0.675	NA	NA	NA	NA
Combined marker (CM)					
CM-2	0.0006	2.7; 1.5–4.8	0.855	91.7	72
CM-6	0.0001	2.7; 1.6–4.5	0.979	100	86
Multivariate analysis					
Marker	*p*-value	Unit HR; 95% CI			
Multivariate analysis with CM-2					
CM-2	0.0016	1.3; 1.1–1.5			
N-stage	0.0217	N2 vs. N0 only: 8.6; 1.7–42.0			
Multivariate analysis with CM-6					
CM-6	<0.0001	1.1; 1.0–1.1			
N-stage (N1 vs. N0)	0.0172	N1 vs. N0 = 3.8; 1.2–12.4 N2 vs. N0 = 8.9; 1.7–46.			

**Table 2:** Univariate and multivariate analysis of clinical parameters and IHC inferences to predict metastasis in cohort-2. Logistic regression analyses were performed on demographic, clinical parameters, and each biomarker. IHC biomarkers showing area under the curve > 0.8 on the ROC curves were combined. The Cox Proportional Hazards Model evaluated the ability of clinical parameters and combined markers to associate with metastasis. The model included age, sex, T-stage, N-stage, perineural invasion (PNI), lymphovascular invasion (LVI), and a combination biomarker. Parameters reaching significance are shown. NA: Not applicable.

Univariate Analysis					
Parameter	*p*-Value	Unit OR; 95% CI	AUC	Sensitivity	Specificity
Age	0.526	NA	NA	NA	NA
Sex	0.23	NA	NA	NA	NA
T-stage	0.0546	NA	NA	NA	NA
N-Stage (+ vs. −)	0.2774	NA	NA	NA	NA
M-stage (+ vs. −)	NA	NA	NA	NA	NA
PNI + vs. −)	0.678	NA	NA	NA	NA
LVI (+ vs. −)	0.304	NA	NA	NA	NA
HYAL-1	<0.0001	23.6; 5.2–107.6	0.944	90	91.7
HA	0.0002	73.5; 7.5–716.8	0.957	90	97.2
HAS2	<0.0001	20.8; 4.7–91.2	0.909	80	97.3
N-Cadh	0.0024	10.3; 2.3–46.7	0.807	85	70.3
Slug	0.0014	3.2; 1.6–6.7	0.757	50	97.3
MMP-9	0.0002	5.4; 2.2–12.8	0.816	80	86.5
Combined marker (CM)					
CM-2 (HYAL-1-HA)	0.0004	2.7; 1.6–4.7	0.986	95	94.6
CM-2 (HYAL-1-HAS-2)	0.0004	2.7; 1.6–4.6	0.984	100	91.9
CM-2 (HYAL-1-N-Cadh)	<0.0001	2.7; 1.6–4.5	0.955	90	94.6
CM-4 (HYAL-1 + HA + Slug + MMP-9)	0.004	2.7; 1.4–5.4	0.99	100	94.6
CM-4 (HYAL-1 + HAS-2 + Slug + MMP-9)	0.003	2.7; 1.4–5.2	0.986	100	91.9
Multivariate analysis					
Marker	*p*-value	Unit HR; 95% CI			
CM-2 (HYAL-1 + HA)	<0.0001	1.6; 1.3–2.0			
CM-2 (HYAL-1 + HAS-2)	<0.0001	1.5; 1.3–1.8			
CM-2 (HYAL-1 + N-Cadh)	<0.0001	1.6; 1.3–2.0			
CM-4 (HA)	<0.0001	1.4; 1.2–1.7			
CM-4 (HAS-2)	<0.0001	1.4; 1.2–1.7			

**Table 3:** Univariate and multivariate analysis of clinical parameters and transcript levels to predict OS in the TCGA COADREAD cohort. Logistic regression analyses were performed on demographic, clinical parameters, and the transcription signatures, CM-2 and CM-6. The Cox Proportional Hazards Model was used to evaluate the ability of clinical parameters and marker signatures to associate with OS; parameters reaching significance are shown. NA: Not applicable.

Univariate Analysis		
Parameter	*p*-Value	Unit OR; 95% CI
Age	0.0008	1.03; 1.01–1.06
Sex	0.503	NA
T-stage (< vs. ≥ 3)	0.01	2.4; 1.2–4.5
N-Stage (+ vs. −)	0.0004	2.5; 1.5–4.1
M-stage (+ vs. −)	0.0005	3.2; 1.7–6.0
CM-2	0.0012	2.7; 1.5–5
CM-6	0.001	2.7; 1.5–4.9
Multivariate analysis with CM-2		
Marker	*p*-value	Unit HR; 95% CI
Multivariate analysis with CM-2		
Age	0.0004	1.04; 1.02–1.07
Sex	0.3959	NA
T-stage	0.005	T4 vs. T2 = 3.6; 1.1–11.7; T4 vs. T3 = 3.3; 1.7–6.5
N-stage	0.0373	N1 vs. N0 = 2.3; 1.1–4.5; N2 vs. N0 = 2.8; 1.4–5.6
M-stage	0.0036	2.5; 1.4–4.7
CM-2	0.0077	2.4; 1.3–4.7
Multivariate analysis with CM-6		
Age	0.0005	1.04; 1.02–1.07
Sex	0.3978	
T-stage	0.0039	T4 vs. T2 3.6; 1.1–11.9; T4 vs. T3 = 3.5; 1.8–6.8
N-stage	0.0339	N1 vs. N0 = 2.3; 1.2–4.5; N2 vs. N0 = 2.9; 1.5–5.6
M-stage	0.004	2.5; 1.3–4.7
CM-6	0.0055	2.4; 1.3–4.4

## Data Availability

All data are provided in this paper. All other data associated with this study are presented in the Supplementary Materials.

## Supplementary Materials

The following supporting information can be downloaded at https://www.mdpi.com/article/10.3390/cancers17060919/s1, Table S1: Patient characteristics in the study cohorts. Table S2: RT-qPCR primer sequences. Table S3: The following antibodies and regents were used for the study. Table S4: Data for calculating combined marker signatures. Table S5: Association of markers and combined biomarker signatures with clinical parameters in cohort-1. Table S6: Univariate and multivariate analysis of combined biomarker signatures to predict OS in the TCGA colon adenocarcinoma (COAD) subgroup dataset. Table S7: Univariate and multivariate analysis of combined biomarker signatures to predict overall survival in the TCGA rectal adenocarcinoma (READ) subgroup dataset. Figure S1: Box plot for the data presented in Figure 1; Figure S2: Uncropped images and densitometric scanning of immunoblot data presented in Figure 3C. Figure S3: Risk stratification of the TCGA COADREAD dataset by CM-2 and CM-6 transcript combination signatures.

## Abbreviations

CRC	Colorectal cancer
HA	Hyaluronic acid
CD44S	CD44 standard
CD44V	CD44 variant
EMT	Epithelial–mesenchymal transition
OS	Overall survival
