# Supplementary material for: Novel Molecular Signatures Selectively Predict Clinical Outcomes in Colon Cancer
Source: Cancers (Basel). 2025 Mar 7;17(6):919. doi: 10.3390/cancers17060919 (PMC11940726; doi:10.3390/cancers17060919)
Supplement: Supplementary file 1 [file cancers-17-00919-s001.zip › cancers-3436041-supplementary.pdf]

Supplementary Information:

Novel Molecular Signatures Selectively Predict Clinical Outcomes in Colon Cancer

Supplementary Table S1: Patient characteristics in the study cohorts. Clinical cohorts 1 and 2: All specimens were deidentified and archival and were obtained from the Biorepository Alliance of Georgia for Oncology (BRAG-Onc). The cohorts shared 38 tumor specimens as both fresh frozen (for RT-qPCR) and FFPE (for IHC) specimen were available from the same patients. Both cohorts included only 2 rectal specimens. In this cohort, patients positive for metastasis had disseminated disease (distant metastasis). TCGA-dataset: The TCGA COADREAD dataset has 383 patients on which transcript expression data are available. The dataset contains patients with COAD (colon adenocarcinoma, n = 245; colon mucinous adenocarcinoma, n = 37), READ (Rectal adenocarcinoma, n=87; rectal mucinous adenocarcinoma, n=5), discrepancy (n=3), and missing histologic type (n=6; anatomic site was listed for two specimens). Follow-up information was not available for time to metastasis; M-stage (synchronous metastasis) data were available.

Parameter	Clinical cohort – 1	Clinical Cohort – 2	TCGA-dataset
Specimens	Normal = 27; Tumor = 67 COAD: 65; READ: 2	Normal = 11; Tumor = 57; COAD: 55; READ: 2	Tumor = 383 COAD: 282; READ: 92; Discrepancy: 3; missing 6
Gender	Male: 37; Female: 30	Male: 28; Female: 29	Male: 208; Female: 171, Missing: 4
Age (years)	Median: 65; Mean: 63.1 ± 12.63	Median: 63.85; Mean: 62.41 ± 10.96	Median: 66; Mean: 64.45 ± 13.08
T-stage	T1: 5; T2: 13; T3: 40; T4: 9	T0 = 3; T1: 2; T2: 12; T3: 34; T4: 6	T1: 10; T2: 57; T3: 260; T4: 49 Missing: 7
N-stage	(-): 33; (+): 34	(-): 34; (+): 23	(-): 207; (+): 168 Missing: 8
LVI	(-): 45; (+): 22	(-): 41; (+): 15 Missing: 1	(-): 230; (+): 102 Missing: 51
PNI	(-): 50; (+): 17	(-): 46; (+): 10 Missing: 1	(-): 169; (+): 58 Missing: 156
M-stage			(-): 255; (+): 51 Missing: 77
Metastasis	(-): 43; (+): 24	(-): 37; (+): 20	
Metastasis (months)	26.91 ± 32.64	40.81 ± 45.43	
Overall survival (OS)	(-): 56; (+): 10 Missing: 1	(-): 52; (+): 5	(-): 285; (+): 86 Missing: 12
Follow up months (OS)	33.91 ± 33.46	49.13 ± 43.73	31.44 ± 27.77
Disease-specific survival			(-): 316; (+): 41 Missing: 26
Follo-up months (DSS)			30.95 ± 27.8

Supplementary Table S2: **RT-qPCR primer sequences.**

Gene	Forward Primer	Reverse Primer
β-Actin	ACTGGAACGGTGAAGGTGAC	AGAGAAGTGGGGTGGCTTTT
HYAL-1	AAGCCCTCCTCCTCCTTAACC	AGCCAGGGTAGCATCGACA
HAS-1	GGTGGGGACGTGCGGATC	CAGGATACACAGTGGAAGTAG
HAS-2	TGAACAAAACAGTTGCCCTTT	TTCCCATCTATGACCATGACAA
HAS-3	CTCTACTCCCTCCTCTATATGTC	AACTGCCACCCAGATGGA
HYAL-4	CATCTGGAAAAAGCTGACCAA	AACATCTTTTGAGTTCCAGTTCC
CD44S	CTGTACACCCCATCCCAGAC	TGTGTCTTGGTCTCTGGTAGC
CD44V	CAGGTGGAAGAAGAGACC CAA	GCTGAGGTCACTGGGATG AA
RHAMM	CAGCTGGAAGATGAAGAAGGA	GCATCTAGTTGTAGCTGAAAAGG
TWIST1	GCGGGAGTCCGCAGTCTTA	TGAATCTTGCTCAGCTTGTC
SNAI1	GAGGCGGTGGCAGACTAG	GACACATCGGTCAGACCAG
N-Cadh	TGGGAATCCGACGAATGG	TGCAGATCGGACCGGATACT
β-Catenin	TGTGGATACCTCCCAAGTCC	TCATTGCATACTGTCCATCAA
Slug	CCCTGAAGATGCATATTCGGAC	CTTCTCCCCCGTGTGAGTTCTA
MMP-9	CTGCCAGGACCGCTTCTACT	CTCAGGGCACTGCAGGATGT
Vimentin	TTTTTCCAGCAAGTATCCAACC	GGAGTTTTCCAAAGATTTATTGAA

Supplementary Table S3: **The following antibodies and regents were used for the study.**

**Target**	**Host**	**Supplier**	**Catalog Number**	**Clone**	**Lot #**	**Blocking & dilution**
**Antibodies for Immunoblotting (4°C overnight)**						
HYAL-1	Rabbit	GeneScript	Custom Synthesized	Polyclonal Aff. purified	1	3% Milk; 1:1,000
HAS-2	Rabbit	GeneScript	Custom Synthesized	Polyclonal Aff. purified	1	3% Milk; 1:1,000
N-Cadh	Rabbit	CellSignaling	4061S	Polyclonal Aff. purified	3	3% Milk; 1:1,000
Slug	Rabbit	Abclonal	A1057	Polyclonal Aff. purified	00022 70201	3% Milk; 1:1,000
MMP-9	Rabbit	Novus	NBP1-57940	Polyclonal Aff. purified	QC49 618-42587	3% Milk; 1:1,000
Actin-HRP	Goat	Santa Cruz	Sc-1615HRP	C11	*J0914	5% NFDM; 1:50,000
**Antibodies for IHC (4°C overnight)**						
Biotinylated HA binding protein	Bovine nasal cartilage	N/A	Custom Synthesized	Clone	1	DAKO antibody diluent; 1:1,000
HYAL-1	Rabbit	GeneScript	Custom Synthesized	Polyclonal Aff. purified	1	DAKO antibody diluent; 1:1,000
HAS-2	Rabbit	GeneScript	Custom Synthesized	Polyclonal Aff. purified	1	DAKO antibody diluent; 1:1,000
N-Cadh	Mouse	Invitrogen	33-3900	3B9	UC284 646	DAKO antibody diluent; 1:50
Slug	Rabbit	Abclonal	A1057	Polyclonal	00022 70201	DAKO antibody diluent; 1:100
MMP-9	Rabbit	Abclonal	A2095	Polyclonal	11516 90301	DAKO antibody diluent; 1:100
**Biological samples**						
Specimens	Source:		Type	Identification		
Cohort-1	Biorepository, BRAG-Onc		Snap frozen	Deidentified		
Cohort-2	Biorepository, BRAG-Onc		Formalin fixed	Deidentified		
TCGA COADREAD	TCGA – Publicly available – Xena Browser download		NA			
**Reagents used**						
Reagent	Supplier	Catalog #				

RNeasy™ Fibrous Tissue Mini Kit	Qiagen	74704
iScript™ cDNA Synthesis Kit	BioRad	1708891
SsoFast™ Evagreen Supermix	BioRad	1725204
DC™ Protein Assay	Bio Rad	5000111
Protease inhibitor cocktail	Millipore Sigma	P8340
Vectastain Elite ABC Kit, Peroxidase (Standard)	Vector Labs	PK-6100
Universal LSAB2 Kit/HRP	DAKO/Agilent	K060911-8
Liquid DAB+ Substrate Chromogen System	DAKO/Agilent	K3467 (K346711-2
Protein block serum-free	DAKO/Agilent	X090930-2

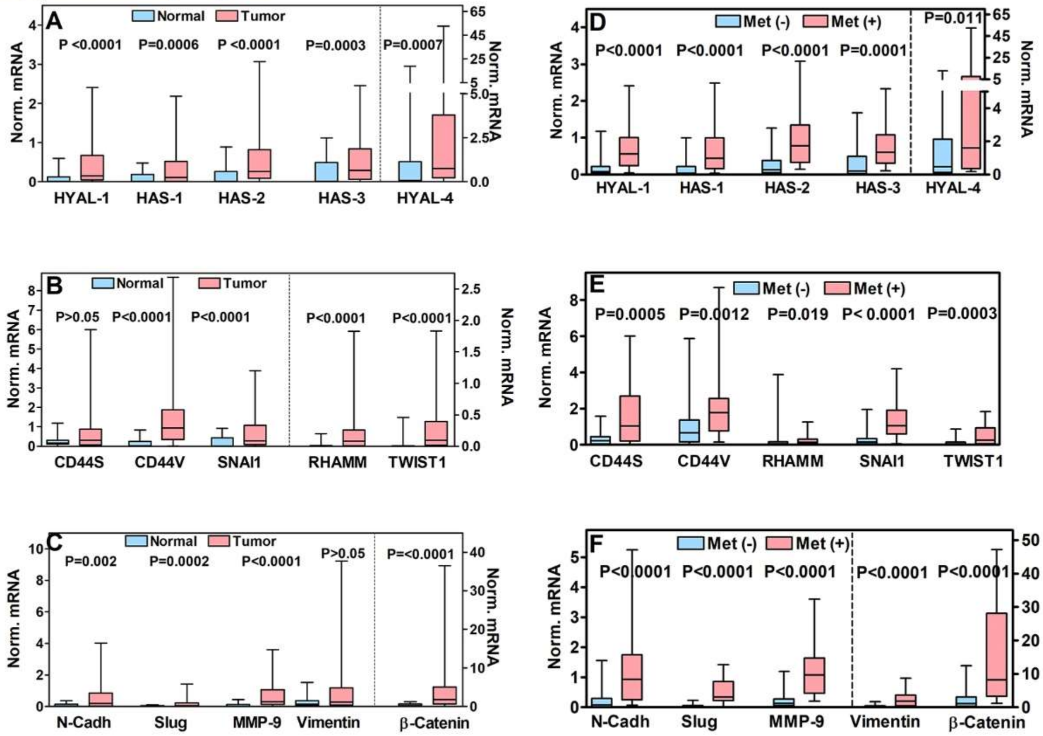

Figure S1: Box plot for the data presented in Figure 1. Data presented in Figure 1 on the differential transcript expression of 15 HA-family and EMT markers in Clinical Cohort 1, were replotted as box plot to show the data spread in a different format.

Supplementary Table S4: Data for calculating combined marker signatures. Combined marker signatures were calculated by performing logistic regression analysis and including the indicated markers. The expression of the signature for each specimen/patient was calculated using the intercept and the coefficient for each marker that was included in the model. The levels of a signature expressed in each specimen were calculated as: 1+(Intercept + α 1*X1+ α 2*X1…). α 1, α 2, etc. are the coefficients for individual markers calculated by the logistic regression model. For clinical cohort 2, the expression was calculated as: (Intercept + α 1*X1+ α 2*X1…).

Cohort-1 – Transcript levels			
CM-2		CM-6	
Intercept	Coefficient α	Intercept	Coefficient α
-1.8387047	HYAL1: 1.4417382<br>N-Cadh: 1.214	-4.5416094	HYAL-1: -2.351296<br>HAS-2: 3.06203866<br>SNAI1: -3.0927779<br>N-Cadh: 3.16970274<br>Slug: 15.4651689<br>MMP9: 2.0923229
TCGA cohort			
CM-2		CM-6	
Intercept	Coefficient α	Intercept	Coefficient α
COADREAD			
0.4316408	HYAL-1: -0.1439322<br>N-Cadh: -0.161253	0.50728637	HYAL-1: -0.1500767<br>HAS-2: -0.0409983<br>SNAI1: 0.00400709<br>N-Cadh: -0.1785772<br>Slug: -0.0140268<br>MMP-9: 0.04088988
Cohort-2 - IHC			
Intercept	Coefficient α	Intercept	Coefficient α
CM-2 (HYAL-1+HA)		CM-2 (HYAL-1+HAS-2)	
-8.2508749	HYAL-1: 1.95749701<br>HA: 3.04990567	-10.142885	HYAL-1: 2.93328659<br>HAS-2: 2.58077104
CM-2 (HYAL-1+N-Cadh)		CM-4 (HYAL-1+HA+Slug+MMP-9)	
-8.0139883	HYAL-1: 3.03520744<br>N-Cadh: 2.04309617	-9.6068621	HYAL-1: 3.3597078<br>HA: 2.59962683<br>Slug: -3.1136528<br>MMP-9: 3.39211926
CM-4 (HYAL-1+HAS-2+Slug+MMP-9)			
-10.59746	HYAL-1: 3.9800836; HAS-2: 1.54154435;<br>Slug: -2.0289408;<br>MMP-9: 2.8181669		

Supplementary Table S5: Association of markers and combined biomarker signatures with clinical parameters in cohort-1. The Mann-Whitney U-test was used to determine the association of individual markers and CM-2 (HYAL-1+N-Cadh) and CM-6 (HYAL-1+HAS-2+N-Cadh+SLUG+SNAI1+MMP-9) signatures with clinical parameters. NS: P-value > 0.05.

	P-value two-tailed				
Marker	T-stage (< or ≥ 3)	N-stage	LVI (+/-)	PNI (+/-)	OS
HYAL-1	NS	NS	NS	NS	NS
HAS-1	NS	NS	NS	NS	NS
HAS-2	NS	0.0027	NS	NS	NS
HAS-3	NS	NS	NS	NS	NS
HYAL-4	NS	NS	NS	NS	NS
CD44S	NS	NS	NS	NS	NS
CD44V	NS	NS	NS	NS	NS
RHAMM	NS	NS	NS	NS	NS
SNAI1	NS	0.0077	NS	NS	NS
TWIST1	NS	0.0194	NS	NS	NS
N-Cadh	NS	0.0404	NS	NS	0.0359
Slug	0.0396	0.0454	NS	NS	NS
MMP-9	NS	0.0025	NS	NS	NS
Vimentin	0.0351	NS	NS	NS	NS
β-Catenin	NS	NS	NS	NS	NS
CM-2	NS	NS	NS	0.026	NS
CM-6	NS	0.0221	NS	0.0104	NS

Raw Data Figure 3C

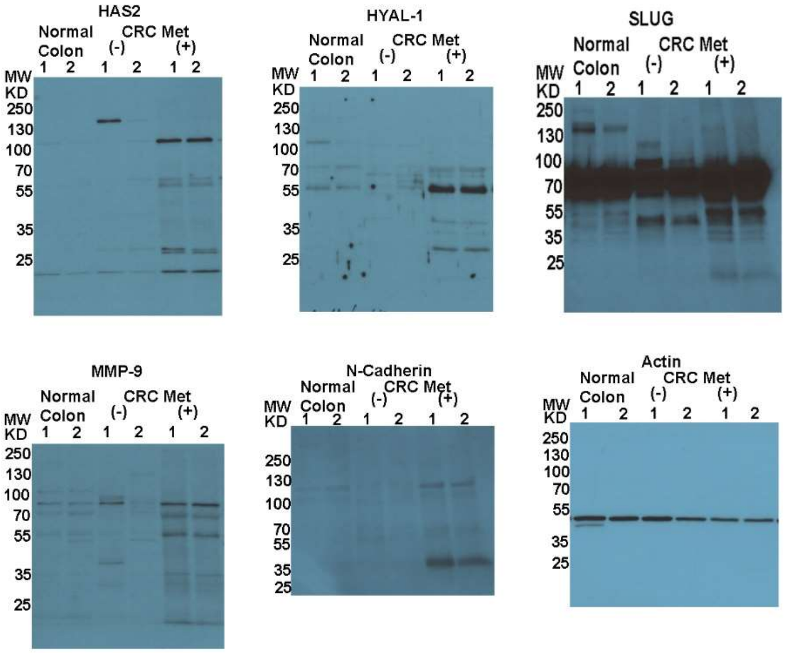

Target	Normal Colon 1	Normal Colon 2	CRC (-) 1	CRC (-) 2	Met (+) 1	Met (+) 2
HAS-2	0.024	0.022	0.012	0.025	1.053	1.027
HYAL-1	0.075	0.061	0.071	0.086	0.834	0.921
MMP9	0.073	0.092	0.827	0.069	1.149	1.080
N-Cad	0.084	0.091	0.105	0.105	1.017	1.054
Slug	0.124	0.080	0.974	0.798	1.239	1.239

Loading Control: Actin

Ratio: Target Protein/Loading Control

Supplementary Figure S2: Uncropped images and densitometric scanning of immunoblot data presented in Figure 3C.

Supplementary Table S6: Univariate and multivariate analysis of combined biomarker signatures to predict OS in the TCGA colon adenocarcinoma (COAD) subgroup dataset. Logistic regression analyses were performed on demographic, clinical parameters, and the transcription signatures, CM-2 and CM-6. The Cox Proportional Hazards Model was used to evaluate the ability of clinical parameters and marker signatures to associate with OS; parameters reaching significance are shown.

Parameter	P-value	Unit OR; 95% CI
CM-2	0.0054	2.6; 1.3 – 5.1
CM-6	0.0045	2.6; 1.4 – 5.2
Multivariate analysis		
Marker	P-value	Unit HR; 95% CI
Multivariate analysis with CM-2		
Age	0.0155	1.03; 1.01 – 1.06
Sex	0.9317	
T-stage	0.0125	T4 vs. T2 = 11; 1.33 – 90.9<br>T4 vs. T3 = 3.3; 1.5 – 7.1
N-stage	0.5195	
M-stage	0.0018	4.0; 2.0 – 8.0
CM-2	0.0238	2.4; 1.1 – 5.2
Multivariate analysis with CM-6		
Age	0.0182	1.03; 1.01 – 1.06
Sex	0.9495	
T-stage (A)	0.0077	T4 vs. T2 = 11.5; 1.4 – 95.3<br>T4 vs. T3 = 3.5; 1.6 – 7.6
N-stage	0.5009	
M-stage	0.0019	3.94; 2.0 – 8.0
CM-6	0.0177	2.4; 1.2 – 4.9

Supplementary Table S7: Univariate and multivariate analysis of combined biomarker signatures to predict overall survival in the TCGA rectal adenocarcinoma (READ) subgroup dataset. Logistic regression analyses were performed on demographic, clinical parameters, and the transcription signatures, CM-2 and CM-6. The Cox Proportional Hazards Model was used to evaluate the ability of clinical parameters and marker signatures to associate with OS; parameters reaching significance are shown.

Parameter	P-value	Unit OR; 95% CI
CM-2	0.3002	
CM-6	0.3394	
Multivariate analysis		
Marker	P-value	Unit HR; 95% CI
Multivariate analysis with CM-2		
Age	0.0017	1.11; 1.04 – 1.17
Sex	0.7218	
T-stage (A)	0.3599	
N-stage	0.120	
M-stage	0.6369	
CM-2	0.5347	
Multivariate analysis with CM-6		
Age	0.0022	1.1; 1.04 – 1.18
Sex	0.7492	
T-stage (A)	0.3893	
N-stage	0.1195	
M-stage	0.6742	
CM-6	0.6529	

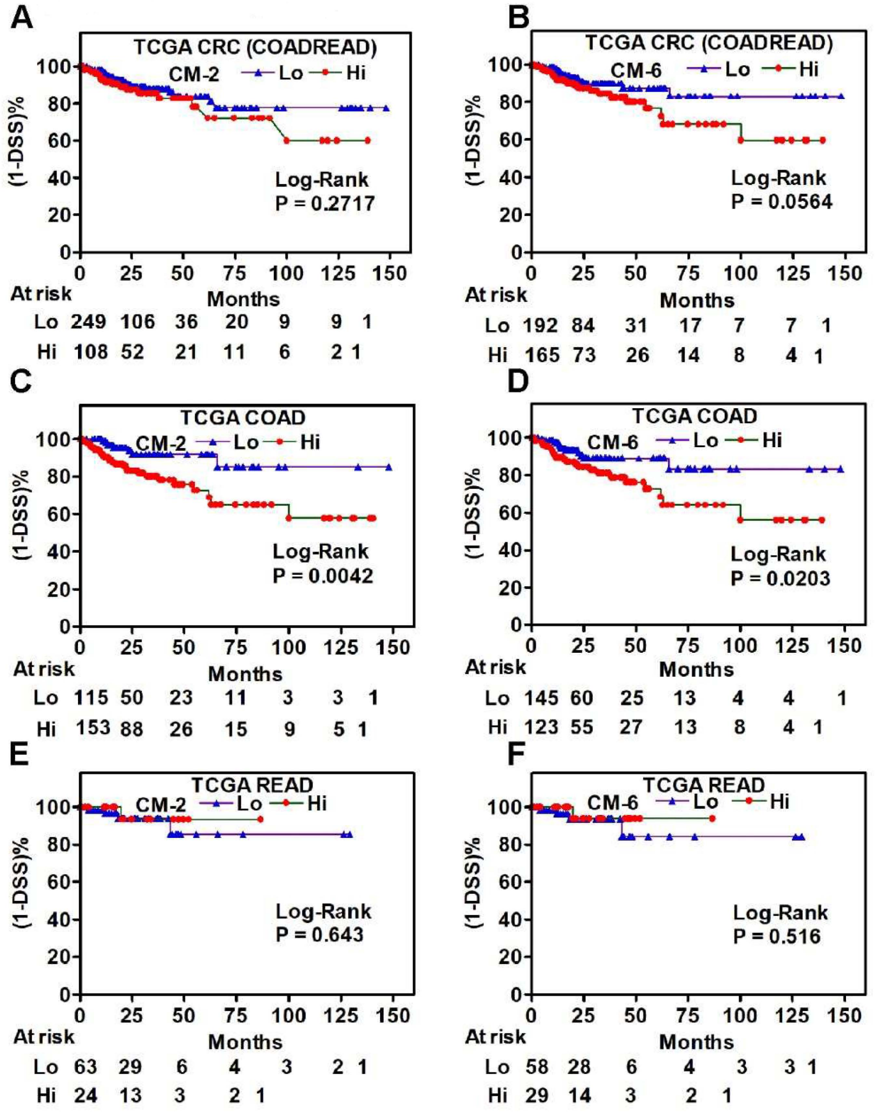

Supplementary Figure S3: Risk stratification of the TCGA COADREAD dataset by CM-2 and CM-6 transcript combination signatures. TCGA COADREAD and COAD and READ subgroups in the TCGA dataset were risk stratified for disease specific survival (DSS) by CM-2 (HYAL-1+N-Cadh) and CM-6 (HYAL-1+HAS-2+N-Cadh+Slug+SNAI1+MMP-9) transcript signatures. **A, B:** COADREAD dataset. **C, D:** COAD subset. **E, F:** READ dataset. P value Log-Rank test.
